# Mind and body: how the health of the body impacts on neuropsychiatry

**DOI:** 10.3389/fphar.2013.00158

**Published:** 2013-12-18

**Authors:** Thibault Renoir, Kyoko Hasebe, Laura Gray

**Affiliations:** ^1^Melbourne Brain Centre, Florey Institute of Neuroscience and Mental Health, University of MelbourneMelbourne, VIC, Australia; ^2^School of Medicine, Deakin UniversityGeelong, VIC, Australia

**Keywords:** mind-body interactions, psychiatric diseases, stress response, HPA axis, mood disorders, peripheral disorders, inflammatory processes

## Abstract

It has long been established in traditional forms of medicine and in anecdotal knowledge that the health of the body and the mind are inextricably linked. Strong and continually developing evidence now suggests a link between disorders which involve Hypothalamic-Pituitary-Adrenal axis (HPA) dysregulation and the risk of developing psychiatric disease. For instance, adverse or excessive responses to stressful experiences are built into the diagnostic criteria for several psychiatric disorders, including depression and anxiety disorders. Interestingly, peripheral disorders such as metabolic disorders and cardiovascular diseases are also associated with HPA changes. Furthermore, many other systemic disorders associated with a higher incidence of psychiatric disease involve a significant inflammatory component. In fact, inflammatory and endocrine pathways seem to interact in both the periphery and the central nervous system (CNS) to potentiate states of psychiatric dysfunction. This review synthesizes clinical and animal data looking at interactions between peripheral and central factors, developing an understanding at the molecular and cellular level of how processes in the entire body can impact on mental state and psychiatric health.

## Introduction

The concept that our mind and our mental processes are influenced by the health of our bodies is intuitively appealing and central to many approaches to health and wellbeing. However, there has been a recent explosion of clinical and physiological evidence to support this theory, shifting a “commonsense” approach to health toward a clinically useful and pharmacologically targetable model. We are now moving toward mechanistic models for the interactions between peripheral and central factors, gaining an understanding at the molecular and cellular level of how processes in the entire body can impact on mental state and psychiatric health. Although good evidence exists for these associations in many psychiatric disorders, in this review we will focus on depression, for which the evidence is perhaps most compelling.

Some epidemiological associations between corporeal disorders and psychological ill-health are well established. The link between coronary artery disease and depression, for example, has been extensively investigated (Nemeroff and Musselman, 2000; Rugulies, 2002; Barth et al., 2004), and it appears that not only are the two disorders strongly associated but that depression is a predictor of poor cardiovascular outcome. Such epidemiological evidence reinforces the widely held notion that the sadness of depression both co-occurs with and potentiates cardiac disease. However, we are now moving toward an understanding of the shared molecular processes which may underpin the link between these disorders.

Although evidence of psychiatric and peripheral comorbidities abounds in the literature, there is also growing interest in the more subtle variations in physiological function which may be antecedents of overt illness but which may be sufficient to modulate CNS processes and mental state. In this review we will focus on several of the major pathways implicated in the aetiology of depression which may mediate the links between the mind and body.

## Systemic disorders associated with depression

Strikingly, a recent study conducted in the United States indicated that of middle aged or older adults meeting diagnostic criteria for a major depressive disorder, two thirds reported comorbid cardiovascular disease (González and Tarraf, 2013). Up to 20% of patients with coronary heart disease meet diagnostic criteria for major depression, and up to 47% report significant and long-lasting depressive symptoms (Bush et al., 2005; Carney and Freedland, 2008). Recent reports have indicated that this effect is not restricted to individuals with cardiovascular disease, as patients undergoing rehabilitation for pulmonary disease were even more likely than cardiac patients to exhibit clinically significant depression and psychological distress (Serber et al., 2012). Cardiovascular risk factors are pathologically relevant even prior to diagnosis. Studies of patients with long-term depressive or anxiety disorders revealed elevated incidence of sub-clinical cardiovascular disease, as measured by a variety of parameters including plaque deposition and arterial stiffness (Seldenrijk et al., 2013), and blood pressure, glucose, body mass index (BMI), diet, and physical activity (Kronish et al., 2012). Interestingly, and relevant to sex differences often observed in the context of anxiety and depressive disorders [for which prevalence can be as twice higher in women compared to men, see Bekker and van Mens-Verhulst (2007); Kimbro et al. (2012)], significant depressive symptoms are more common in younger women with peripheral arterial disease than in other gender-age groups (Smolderen et al., 2010). Also, recent meta-analysis of cardiovascular risk factors and depression in later life demonstrated relatively strong associations between depression and diabetes, cardiovascular disease and stroke (Valkanova and Ebmeier, 2013).

These findings also highlight the relationship between diabetes and psychiatric health. Meta-analytic evidence suggests that patients with depression have an elevated risk of developing type two diabetes (Knol et al., 2006; Mommersteeg et al., 2013), and conversely that patients with diabetes have significantly increased risk of developing depression (Anderson et al., 2001; Rotella and Mannucci, 2013). A longitudinal study revealed that the incidence of diabetes was highest in individuals with the greatest number of depressive symptoms (Carnethon et al., 2003), and a large community-based study demonstrated that diabetes was associated with an increased risk of depression (de Jonge et al., 2006). This bi-directional relationship is suggestive of convergent pathological processes rather than a simplistic cause and effect relationship. Interestingly, some clinical studies have hypothesized that the doubled rates of depression in female diabetic patients could help explain the high prevalence of coronary heart disease in women with diabetes (Clouse et al., 2003).

Autoimmune disease, for example rheumatoid arthritis, is also associated with markedly elevated risk of depression (Margaretten et al., 2011a; Covic et al., 2012). Notably, there appears to be a strong correlation between the severity of rheumatoid arthritis and the incidence of depression, with a recent meta-analysis demonstrating that those with the most severe form of arthritic disease have a six-fold higher incidence of depression relative to those with the mildest form Godha et al. (2010).

Clearly the impact of declining quality of life associated with severe systemic disease cannot be overlooked. However, these findings and the many others describing strong associations with psychiatric disease and peripheral illness do provoke the question of whether there are fundamental mechanisms in common. How does the health of the body affect the health of the mind, and what are the underlying pathological processes which underpin this relationship? Although we do not yet have a full understanding of the complexities of the bidirectional relationship between body and brain, convergent evidence suggests that the endocrine response to stress (via the HPA axis), and immune dysregulation (via inflammatory pathways), may be playing a central role.

## Stress responsivity and the hypothalamic-pituitary-adrenal axis

The most well established example of mind-body interaction is the link between psychological stress and psychological ill-health. In fact, adverse or excessive responses to stressful experiences are built into the diagnostic criteria for several psychiatric disorders, including depression and anxiety disorders. The body's response to stress is mediated by the hypothalamic-pituitary-adrenal (HPA) axis, by which stressful stimuli modulate the activity of a tightly regulated cycle of circulating hormones. Stress *per se* is not necessarily problematic; the body is well equipped to respond to stressful stimuli and to some extent stress is necessary for normal function. However, excessive or prolonged stress, or perturbations in the function or regulation of the HPA axis may result in abnormal changes in hormones circulating through both the periphery and the CNS. As previously mentioned, women are twice as likely as men to suffer from stress-related psychiatric disorders and there is evidence that sex differences in stress responses could account for this sex bias (Bangasser and Valentino, 2012).

The HPA axis is the primary circuit that mediates the physiological response to stress and regulates the level of circulating glucocorticoid hormones (e.g., CORT: cortisol in humans, corticosterone in rodents). Arginine vasopressin (AVP) and corticotrophin-releasing hormone (CRH, also originally referred to as CRF for corticotrophin-releasing factor) are synthesised and released from the paraventricular nucleus (PVN) of the hypothalamus, and are arguably the highest order regulators of the HPA axis activity within the central nervous system (CNS). These neuro-hormones act synergistically to stimulate adrenocorticotrophin (ACTH) secretion from the anterior pituitary, culminating in increased levels of circulating CORT. The HPA axis is modulated by a negative feedback loop encompassing the hippocampus, hypothalamus and anterior pituitary. Following CORT secretion into the peripheral blood circulation, CORT passes through the plasma membrane of cells, particularly in the pituitary, hypothalamus, and hippocampus where it binds to the glucocorticoid receptor (GR). Finally, glucocorticoid catabolism involves 5α-reductase type 1 (predominantly a liver enzyme) and 11β-hydroxysteroid dehydrogenase type 2 (in kidney).

The psychological determinants of an individual's response to stress are important predictors of outcome, although this area is beyond the scope of this review [reviewed comprehensively by Liu and Alloy (2010)]. However, physiological variations in HPA axis function and related pathways may also modulate the response to stress and alter the threshold for psychiatric disorders. Despite substantial limitations in the objective assessment of stress, multiple studies have documented an association between stressful life experiences and depression (Kendler and Gardner, 2010). Interesting examples of HPA axis dysfunction modulating psychiatric health come from Cushing's syndrome and Addison's disease, states of hyper- and hypo-cortisolemia, respectively. Cushing's syndrome is associated with a high prevalence of psychopathology, primarily depressive symptoms but also mania and anxiety (Pereira et al., 2010). Addison's disease has been less extensively investigated but appears to be associated with an increased risk of a variety of psychiatric symptoms, including depression, delusions, hallucinations, and anxiety (Anglin et al., 2006). In both disorders it should be borne in mind that adrenal dysfunction can also lead to electrolyte and metabolic abnormalities which can also contribute to CNS disturbances. Nonetheless, the fact that treatment of the hyper- or hypo-cortisolaemia resolves the psychiatric symptoms in most cases strongly suggests that changes in adrenal corticosteroids are a primary driving force for the psychiatric symptoms (even though this is not the sole determining factor, as half of subjects with Cushing's do not develop depressive symptoms). Therapeutic administration of high doses of corticosteroids has been associated with the development of a manic behavioral state (Warrington and Bostwick, 2006; Kenna et al., 2011; Fardet et al., 2012). These observations also highlight a critical pathway by which HPA axis function may alter mental state. Corticosteroids are generally prescribed in cases of uncontrolled inflammatory disease, and act as powerful anti-inflammatory factors. As we will discuss below, inflammatory states are strongly linked to perturbations in psychiatric health. More subtle variations in HPA axis function have been directly associated with psychiatric disorders, in particular depression. A recent meta-analysis described the magnitude of the difference between depressed and non-depressed group in cortisol, ACTH and CRH levels. Looking at 361 studies, the results show that overall depression is associated with small-to-moderate elevations in ACTH and cortisol and a reduction in CRH levels (Stetler and Miller, 2011). However, in older people, the association between cortisol and major depression was U-shaped (Bremmer et al., 2007). Another large cohort study revealed significant associations between major depressive disorders and specific HPA axis indicators, such as a higher cortisol awakening response in MDD patients compared to controls (Vreeburg et al., 2009). Those modest but significant differences were also observed in patients with anxiety disorders (Vreeburg et al., 2010).

In line with clinical findings, the circadian pattern of corticosterone has been reported to be disrupted in rodent models of depression (Touma et al., 2009; Bonilla-Jaime et al., 2010). In rats, chronic stress induces a depressive-like phenotype, associated with dysregulation of the HPA axis and reductions in dopaminergic and serotonergic transmissions in the PFC (Mizoguchi et al., 2008). Affective-like behavioral deficits have been reported in mouse mutants with altered HPA axis function [see Renoir et al. (2013) for review]. Chronic treatment with corticosterone as well as isolation rearing increase the depressive-like behavior in GR-dependent and independent manners (Ago et al., 2008). Chronic elevation of corticosterone creates a vulnerability to a depression-like syndrome that is associated with increased expression of the serotonin synthetic enzyme tryptophan hydroxylase 2 (tph2), similar to that observed in depressed patients (Donner et al., 2012). Interestingly, the effects of chronic corticosterone administration in animal models have also been studied in the context of affective and systemic disorders. In that regard, chronic corticosterone in mice was found to induce anxiety/depression-like behaviors (David et al., 2009) as well as decrease sucrose consumption in a model of anhedonia (Gourley et al., 2008). Chronic antidepressant treatment reversed those behavioral impairments. Furthermore, relevant to the relationship between stress and metabolic syndrome, 4-wk exposure to high doses of corticosterone in mice, has been found to increase weight gain and plasma insulin levels as well as reduce home-cage locomotion (Karatsoreos et al., 2010).

Using a chronic mild stress (CMS) paradigm, in which mice were housed individually and alternatively submitted to unpredictable “mild” stressors (such as periods of continuous overnight illumination, short periods of food/water deprivation etc.), Palumbo et al. (2010) found that mice subjected to the CMS procedure exhibited an increase in serum corticosterone levels during the first few weeks of exposure. However, these elevated corticosterone levels returned to baseline levels after 6 weeks of CMS. Similarly, Adzic et al. (2009) reported reduced CORT levels in chronically isolated rats (for 21 days), whereas CORT was increased after an acute 30-min immobilization stress. Altered circadian activity of the HPA axis has also been reported in a CMS rat model of depression (Christiansen et al., 2012). Interestingly, this study suggests a recovery of diurnal corticosterone rhythm after 8 weeks of CMS. Taken together, these observations suggest an adaptive capacity for the HPA axis to cope with prolonged stress.

The effects of chronic stress on HPA axis function have been widely studied in both animal models and clinical populations. Many of those investigations have focused on the negative feedback part of the HPA axis (mainly mediated by the GR). Such feedback is efficiently probed by the established combined dexamethasone-suppression/corticotrophin-releasing hormone stimulation (dex/CRH) test (Ising et al., 2007). Altered dex/CRH test are seen in major depression (Mokhtari et al., 2013) as well as in chronic stress conditions. For example, over-commitment in chronically work-stressed teachers was significantly associated with blunted response to the dex/CRH challenge (Wolfram et al., 2013). Further regression analyses showed that low social support at work and high job strain were associated with more cortisol suppression after the dexamethasone suppression test (Holleman et al., 2012). In rodents, social isolation decreased the feedback sensitivity of the HPA axis to dexamethasone (Evans et al., 2012). Another animal study reported that socially deprived mice had increased adrenal weights as well as a greater increase in corticosterone levels in response to acute stress (Berry et al., 2012). Interestingly, those chronic stress-induced HPA axis dysfunctions were associated with depressive/anxiety-like behavior as well as impaired hippocampal plasticity (i.e., altered hippocampal neurogenesis and reduction in BDNF levels) (Berry et al., 2012; Evans et al., 2012).

Polymorphisms in genes controlling the activity of the HPA axis are also associated with differential risk of psychiatric disease. Polymorphisms in the GR gene have been associated with major depression in multiple cohorts (van West et al., 2005; van Rossum et al., 2006) [but also see Zou et al. (2010); Zimmermann et al. (2011)]. Interestingly, some GR polymorphisms are also a predictor of the HPA axis response to psychosocial tests (Kumsta et al., 2007) and have been found to be associated with the extent of stress hormone dysregulation in major depression (Menke et al., 2013). Genotype-phenotype associations have also been identified in terms of response to antidepressant response (Ellsworth et al., 2013). Evidence of gene-environment interactions in the stress response and psychiatric susceptibility comes from a study of the corticotrophin-releasing factor receptor (CRF-R) (Bradley et al., 2008). Individuals with a particular CRF-R genotype who had experienced child abuse had enhanced risk of depression as adults, an observation repeated in two ethnically different populations. Overall, studies suggest that the degree of HPA axis hyperactivity can vary considerably across psychiatric patient groups, likely due to genetic and environmental factors during early development or adult life. In that regard, two separate studies reported that polymorphisms of the FKBP5 gene that potentially modify the sensitivity of the GR are associated with an increased likelihood of adult depression for individuals exposed to adverse life events (Zimmermann et al., 2011) and childhood physical abuse (Appel et al., 2011). Genes involved in other pathways may also potentiate an aversive response to stress. A landmark early study described an association between a variant in the serotonin transporter gene and the response to stressful life experiences (Caspi et al., 2003). This functional variant in a major target of antidepressant therapies is associated with an elevated response to fearful stimuli, elevated hormonal responses to stress, and increased risk of depression in response to stress exposure (Lesch et al., 1996; Hariri et al., 2002; Jabbi et al., 2007). Variants in multiple genes in the serotonergic pathway have also been associated with altered behavioral phenotypes in animal models [reviewed in Holmes (2008)]. Critically, changes in circulating corticosteroids can regulate the activity of the rate-limiting serotonin synthetic enzyme tryptophan hydroxylase 2 in the brain (Clark et al., 2005, 2007). In rodent models, acute restraint stress up-regulates serotonin production in the amygdala (Mo et al., 2008), whilst chronic administration of ACTH to disrupt HPA axis function results in an increased level of serotonin in the prefrontal cortex in response to acute stress (Walker et al., 2013). Taken together these findings demonstrate that alterations in HPA axis function can directly impact on CNS systems known to be associated with psychiatric disease.

## Peripheral disorders associated with HPA changes and psychiatric disease

A wealth of evidence is now emerging to illustrate the link between stress and risk factors for physiological disorders, in particular metabolic disorders. Hyperactivity of the HPA axis and hypercortisolaemia is associated with the metabolic syndrome (Anagnostis et al., 2009). Similarly, both chronic stress and chronic treatment with glucocorticoids are associated with central adiposity, dyslipidaemia, atrophy of skeletal muscles, insulin resistance, and glucose intolerance: a suite of symptoms remarkably resemblant of the metabolic syndrome itself (Kyrou and Tsigos, 2009; van Raalte et al., 2009).

Elevations of circulating glucocorticoids have also been linked with an increased risk of depression in those with metabolic disorder (Vogelzangs et al., 2009), and relative insensitivity to the dexamethasone suppression test has been documented in patients with this disorder (Kazakou et al., 2012). On the other hand, disturbances in fatty acid metabolism have been observed in cohort studies of depression (Assies et al., 2010). Fatty acid levels appear to have a bidirectional relationship with HPA axis activity, with glucocorticoids modulating fatty acid metabolism (Brenner et al., 2001; Macfarlane et al., 2008), and supplementation of polyunsaturated fatty acids reducing cortisol levels in both healthy subjects (Delarue et al., 2003) and in those with depression (Jazayeri et al., 2010; Mocking et al., 2012). A study examining this relationship in more detail has shown that the circadian changes in cortisol have a different association with the major fatty acid forms in major depression patients compared to controls (Mocking et al., 2013). Other studies have demonstrated both changes in visceral fat levels and adrenal gland volume in women with major depressive disorders (Ludescher et al., 2008). Some of these associations appear to have developmental antecedents, with exposure to dietary high fat in the perinatal period being linked with both altered HPA axis function and mood changes (Sasaki et al., 2013).

If metabolic disorders are considered as a spectrum, then diabetes is arguably positioned as the end point of this decline in function. Chronic stress and sustained dysregulation of corticosteroid production are strongly associated with the development of type 2 diabetes mellitus in both human cohorts and in animal models (Chan et al., 2003; Rosmond, 2005; Reagan et al., 2008; Anagnostis et al., 2009; Matthews and Hanley, 2011). As an example in mice, streptozotocin (STZ)-induced diabetes resulted in increased depressive-like behavior as well as increased corticosterone levels (Ho et al., 2012). The convergence of the associations between HPA axis dysfunction and both diabetes and depression is striking, with compelling evidence for links between the two disorders and this central underlying risk factor [reviewed in Champaneri et al. (2010)].

Dysfunction of HPA signaling also appears to interact with the autonomic nervous system to influence cardiovascular function. Components of the HPA axis act outside the hypothalamus to regulate sympathetic outflow, and thus heart rate. Elevated heart rate has been associated with depression in multiple studies (Forbes and Chaney, 1980; Carney et al., 1993, 2000; Lechin et al., 1995), and is a strong predictor of multiple parameters of cardiovascular disease, including myocardial ischaemia, arrhythmias, hypertension, and cardiac failure (Dyer et al., 1980; Kannel et al., 1987; Palatini and Julius, 1997). Depression is associated with an increased risk of mortality in patients with cardiovascular disease (Mann and Thakore, 1999), and this increased risk is strongly linked with hypercortisolaemia (Jokinen and Nordstrom, 2009). In healthy subjects, cortisol and ACTH response to the Dex/CRH test were negatively associated with central adiposity and blood pressure and positively associated with HDL cholesterol, strong risk factors for cardiovascular disease (Tyrka et al., 2012).

Taken together, these studies speak to the accumulating evidence suggesting a link between disorders which involve HPA dysregulation and the risk of developing psychiatric disease. This is illustrative of the bidirectional relationship between peripheral illness and mental health: HPA axis changes may be either contributors to or consequences of peripheral disorders but also have the capacity to modulate brain function and predispose to psychiatric disease.

## Pharmacological targeting of the HPA axis

The GR antagonist mifepristone has been tested as an adjunctive treatment for psychiatric disorders (Schatzberg and Lindley, 2008). Most recently, a randomized controlled trial of adjunctive mifepristone in patients with bipolar disorder demonstrated alterations in cortisol levels which were correlated with improvements on neuropsychological tests of working memory (Watson et al., 2012). An earlier, smaller scale trial by the same group showed improvements in both neurocognitive function and depression rating scores (Young et al., 2004). However, a similar study in schizophrenia showed alterations in plasma cortisol but no significant change in symptoms (Gallagher et al., 2005). These mixed findings do highlight the potential utility of therapeutics targeting HPA axis function, but also are suggestive of the heterogeneity in the role of the HPA axis across, and potentially also within, psychiatric disorder diagnoses. The main challenge in pharmacological targeting of the HPA axis is that blockage of all GR-dependent processes could ultimately lead to counteractive effects such as elevated endogenous corticosterone levels. In that context, a newly developed high-affinity GR ligand (C108297) shows promising characteristics in rats (Zalachoras et al., 2013). Indeed, C108297 displays partial agonistic activity for suppression of CRH gene expression and potently enhances GR-dependent memory consolidation. This compound, which does not lead to disinhibition of the HPA axis, could help in dissecting the molecular signaling pathways underlying stress-related disorders. In recent years, other therapeutic strategies interacting at different levels of the HPA axis have been developed. Those include agents acting on CRH-R1 receptor and adrenal steroidogenesis as well as modulators of the 11β-hydroxysteroid dehydrogenase type (11β-HSD1), the enzyme regulating cortisol metabolism (Thomson and Craighead, 2008; Martocchia et al., 2011).

In patients who were successfully treated with fluoxetine, the secretion of cortisol decreased (Piwowarska et al., 2012). Furthermore, recent data suggest that GR levels in lymphocytes could be used to predict response to antidepressant treatment in major depressive patients (Rojas et al., 2011). However, it should be noted that GR levels seemed inconsistent over time in this study. Also, measuring cortisol levels in depressed patients before and following treatment with SSRI, Keating et al. (2013) concluded that that stress physiology was unlikely to be a key factor in the response to antidepressant treatment. The variation in findings from these studies may reflect differing modes of activity of the different antidepressant drug classes, superimposed on a heterogeneous patient population. This was illustrated in a study examining changes in daily cortisol patterns in patients using SSRIs, tricyclic antidepressants, other therapeutics or no medications (Manthey et al., 2011). A complex pattern emerged, with some antidepressants suppressing the morning peak in cortisol, and others altering the response to the dexamethasone suppression test. However, the challenges inherent to measuring a circulating factor which is both diurnally regulated and acutely sensitive to environmental cues should not be underestimated.

## Immune dysregulation, inflammation and psychiatric health

There is strong evidence that peripheral growth factors, pro-inflammatory cytokines, endocrine factors, and metabolic markers contribute to the pathophysiology of major depressive disorders and antidepressant response (Schmidt et al., 2011). Similarly, many of the systemic disorders associated with a higher incidence of psychiatric disease involve a significant inflammatory component. In fact, as our understanding of the aetiology of these disorders deepens, it has become apparent that there is significant overlap between the factors driving peripheral inflammatory disease and psychiatric disorders. Elevations of pro-inflammatory cytokines have been observed in both clinical populations and animal models of heart failure (Levine et al., 1990; Francis et al., 2003), after coronary surgery (Hennein et al., 1994), and following heart transplants (Azzawi and Hasleton, 1999). Importantly, the pathogenesis of atherosclerosis is intrinsically inflammatory (Koenig, 2001), with elevated local and circulating pro-inflammatory cytokines. In addition, the acute-phase marker C-reactive protein (CRP) is strongly associated with cardiovascular disease (van Holten et al., 2013), and can be used as a diagnostic or prognostic factor. As discussed above, cardiovascular disease is strongly associated with changes in psychiatric health, in particular depression.

Cardiovascular disease is in turn closely linked with obesity, dyslipidemia, diabetes and metabolic disease. The elevated frequency of anxiety and depression in these disorders may in part underlie the association between cardiovascular and psychiatric risk factors. In studies of diabetic patient cohorts, the inflammatory marker CRP was consistently predictive of direct associations between depression severity, lipid profiles and obesity levels (van Reedt Dortland et al., 2013). Similarly, increased risk of depression in a cohort of patients with diabetes was associated with a higher BMI, illustrating the link between depression and poor control of cardiovascular risk factors (Kimbro et al., 2012). Obesity itself is considered to be a state of low-grade inflammation, and is linked with elevated depressive symptoms. In addition, in a longitudinal study CRP levels at baseline were statistically associated with depression scores (Daly, 2013).

Other disease states involving inflammatory processes are associated with elevated risk of depression. Major depression is the most common psychiatric manifestation of multiple sclerosis, with an incidence approaching 50% (Lo Fermo et al., 2010). Likewise, although the incidence rate varies significantly between studies, an elevated incidence of depression has been documented in systemic lupus erythematosus (Palagini et al., 2013) and rheumatoid arthritis (Dickens et al., 2002). Common to all of these disorders is an autoimmune-mediated elevation of inflammatory signaling, with increased circulating pro-inflammatory cytokines observed in the periphery and in the CNS. Large case-control studies have described increased rates of anxiety and depression in patients with inflammatory bowel disease (Kurina et al., 2001; Ananthakrishnan et al., 2013a,b). Altered gut permeability to enteric bacteria has also been associated with depression. Translocation of bacterial allergens [in particular lipopolysaccharide (LPS)], stimulates a systemic immune response characterized by elevated IgM and IgA antibodies reactive to the bacteria. Individuals with chronic depression are more likely to display increased LPS-reactive IgM and IgA than control subjects, indicating that elevated gut permeability may be potentiating a systemic inflammatory state (Maes et al., 2008, 2012a).

The case for altered peripheral inflammation in psychiatric disease is strong, perhaps most so for major depression. Individuals with clinically classifiable major depression exhibit a wide range of changes in inflammatory markers, including elevated cytokines, chemokines, and acute phase proteins, findings which have been replicated in several meta-analyses and which in some studies appear to be correlated with specific depressive symptoms (Miller et al., 2009). There appears to be a shift in the function of the immune system in depression, with an increase in pro-inflammatory cytokines accompanied by a decrease in cellular immunity (Zorrilla et al., 2001; Dowlati et al., 2010). The strength of these findings is heightened by a positive correlation between the elevations in pro-inflammatory cytokines and the severity of depression rating scores (Howren et al., 2009). A recent longitudinal population-based study demonstrated strong associations between depressive symptoms and elevated levels of the pro-inflammatory cytokine IL-6 and CRP (Lu et al., 2013). Notably, heightened IL-6, CRP and depressive symptoms were all predictive of reduced pulmonary function, in a cohort with no known history of obstructive pulmonary disease. This large study highlights the substantial cross-over between inflammatory disease and depressive symptomatology. However, there may be differences between sub-populations in depression, with some individuals more likely to display an inflammatory pathophysiology. Although the number of patients classified as suffering atypical depression is relatively low, these patients may be more likely to show high levels of inflammatory markers such as CRP (Hickman et al., 2013). Part of the population variance may result from polymorphisms in the CRP gene. The association between CRP levels and depressive symptoms may be moderated by CRP gene haplotype, in a complex manner which may underpin some of the variations in other association studies (Halder et al., 2010). Patients receiving therapeutic administration of cytokines for cancer or chronic viral infections, [in particular interferon (IFN)-alpha and interleukin-2] frequently experience psychiatric symptoms, including the development of frank major depression in a significant proportion of patients (Capuron et al., 2004; Raison et al., 2005). IFN-alpha stimulates both peripheral and central release of pro-inflammatory cytokines, a fact which underpins the behavioral effects of this cytokine and highlights the capacity for systemic immune signals to regulate CNS processes (Capuron et al., 2000, 2001, 2002, 2003, 2004; Raison et al., 2005; Eller et al., 2009; Alavi et al., 2012; Birerdinc et al., 2012; Udina et al., 2012).

Of particular relevance to the treatment of depressive disorders is the emerging evidence that at least part of the therapeutic efficacy of currently available antidepressants may result from their concomitant anti-inflammatory effects. Although the response rate and efficacy of current antidepressants is far from universal, at least some patient populations derive significant benefit from these medications. However, the previously accepted notion that modulation of synaptic monoamines represents the sum total of the therapeutic effects of these drugs has now come into question. Recent studies have shown that selective-serotonin-reuptake inhibitor medications can suppress immune cell activation and release of inflammatory cytokines in the periphery and *ex-vivo* (Diamond et al., 2006; Taler et al., 2007; Branco-de-Almeida et al., 2011). Notably, this immune-regulatory effect is not restricted only to the periphery, but can also affect microglia, the immune cells of the CNS (Hashioka et al., 2007; Horikawa et al., 2010). A recent meta-analysis of human depression studies showed that antidepressant treatment at least partially ameliorates the elevations of pro-inflammatory cytokines associated with the disorder (Hannestad et al., 2011). Although it is clear that drug discovery in psychiatric disease needs to look beyond established drug classes, these findings emphasize the potential clinical utility of targeting inflammatory function in depression. Finally, potential sex-differences have been suggested when assessing the effects of LPS on cytokine gene expression. Indeed, females had increased hippocampal levels of IL-6 of TNF-α with respect to males after repeated administration of LPS (Tonelli et al., 2008).

## Mechanisms of immune modulation of psychiatric function

Historically the CNS was regarded as a “privileged” site with regards to the immune system, with little immune communication across the blood-brain barrier except in cases of frank CNS infection. However, it is now clear that the brain is sensitive to peripheral immune stimuli and can respond with activation of central immune cells and local production of inflammatory cytokines. Microglia are the CNS equivalent of macrophages, releasing cytokines upon activation and facilitating a central immune response, even in the absence of peripheral immune cell migration into the CNS. The brain's response to peripheral inflammatory stimuli can be seen most clearly in the pattern of behavioral changes which reliably results from systemic infection, administration of synthetic bacterial wall components or administration of cytokines (Dantzer, 2004; Pucak and Kaplin, 2005). Termed “sickness behavior,” this encompasses changes in motor activity, consummatory behavior, social interaction, circadian rhythms, and responsivity to hedonic and aversive stimuli. The parallels between these behavioral changes and aspects of depression have been well noted and have been a prompt for extensive research.

Systemic administration of synthetic bacterial endotoxin, or LPS, induces a well-established pattern of peripheral inflammation. However, multiple studies have now also demonstrated that systemic inflammation activates CNS microglia, including in non-human primates (Henry et al., 2008; Hannestad et al., 2012). In mice, systemic LPS causes microglial activation and synthesis of cytokines (Puntener et al., 2012). Microglia form close contacts with synaptic structures and appear to regulate synaptic strength (Wake et al., 2009). These cells also express multiple neurotransmitter receptors and are therefore acutely responsive to neuronal signaling (Kettenmann et al., 2011). Activated microglia are also a key source of reactive oxygen species, contributing to a status of inflammation-induced oxidative stress in the CNS (Dringen, 2005). Oxidative stress, driven both peripherally and centrally, is strongly associated with psychiatric aetiology.

Reduced plasma L-tryptophan, the precursor for serotonin, is a potential biomarker of “vulnerability to depression” (Maes et al., 1993). Indeed, tryptophan depletion is widely used to study the contribution of reduced serotonin transmission to the pathogenesis of major depressive disorder (Van der Does, 2001) and also relevant in the context of immune activation (Kurz et al., 2011). The depressive symptomatology associated with immunomodulatory therapy may be mediated in part by changes in tryptophan metabolism. Pro-inflammatory cytokines such as IFN-γ, IFN-α, and TNF-α, and reactive oxygen species, induce activation of the enzyme, indolamine 2, 3 dioxygenase (IDO) in microglia, which metabolizes tryptophan via the kyneurenine pathway (Maes, 1999; Wichers et al., 2005; Dantzer et al., 2008; Maes et al., 2012b). This shifts the balance of tryptophan toward kynurenine and away from serotonin, reducing serotonin bioavailability (Capuron et al., 2002, 2003; Vignau et al., 2009). Notably, in the CNS only microglia further metabolize kynurenine to quinolinic acid, which exerts neurotoxic effects (Guillemin et al., 2005; Soczynska et al., 2012). Patients treated with IFN-α for hepatitis C infection developed depressive symptoms including negative moods that were correlated with increased levels of kynurenine (Wichers et al., 2005). In addition, analysis of plasma tryptophan and kynurenine pathway metabolites in patients with major depression showed increased rates of tryptophan degradation compared to normal control subjects (Myint et al., 2007). Taken together, these findings indicate that cytokine-induced microglial activation can mediate changes in neurotransmitters and other bioactive metabolites which may underpin mood disorders. Also, recent data indicate that cognitive impairments (as well as the decline in neurogenesis observed during ageing) can be in part attributed to dysregulation in blood-borne factors such as changes in peripheral CCL11 chemokine levels (Villeda et al., 2011). These findings support the crosstalk between peripheral molecular processes to central effects related to cognitive and emotional function.

## Pharmacological targeting of inflammatory pathways

Several of the therapies for the inflammatory disorder rheumatoid arthritis potentiate the effects of antidepressant therapies (Margaretten et al., 2011b). Such drugs target pro-inflammatory cytokine pathways, for example TNF-α antagonists such as etanercept. This particular drug is also commonly used in the treatment of the inflammatory skin condition psoriasis, and large-scale studies of this drug have indicated that patients with psoriasis receiving this drug show reduced depression scores relative to placebo (although the level of depressive symptoms in these patients was relatively low overall, and would not constitute a diagnosis of major depression) (Tyring et al., 2006). Interestingly, follow-up studies indicated that the change in depression score was independent of disease state (Krishnan et al., 2007). Drugs with a similar TNF-α antagonist activity have also shown antidepressant activity in trials in patients with other inflammatory conditions, including Crohn's disease and ankylosing spondylitis (Persoons et al., 2005; Ertenli et al., 2012).

Critically, a recent study of the TNF-α antagonist infliximab in otherwise healthy patients with major depression demonstrated that the antidepressant activity of this drug was dependent on the level of inflammatory markers at baseline (Raison et al., 2013). This study demonstrated that depressed patients with higher levels of the inflammatory markers TNF-α and CRP showed a decrease in depression rating scores over the course of the study. It is also worth noting that the patients in this study were poorly responsive to classical antidepressant therapy, which may indicate that a sub-population exists in whom inflammation is correlated with both poor antidepressant response and efficacy of anti-inflammatory medication. A second recent study also demonstrated that patients with depression who experienced a decline in symptoms with infliximab treatment also showed elevated inflammatory gene expression in peripheral immune cells (Mehta et al., 2013). Response to infliximab was also associated with reductions in the expression of other genes involved with innate immune activation. Agents such as infliximab are too large to cross the blood-brain barrier, and therefore the amelioration of depressive symptoms is more likely associated with resolution of peripheral inflammation than direct effects of the drug in the brain. However, as we have discussed above, CNS microglia are acutely sensitive to circulating cytokine levels and so their level of activity may well be modulated by anti-inflammatory treatment.

The developing focus on inflammatory function in depression has spurred trials of other anti-inflammatory drugs as adjuncts to antidepressant treatment. A large-scale longitudinal population study revealed that statin users were less likely than non-users to have depression at baseline (Otte et al., 2012). Statin users who did not have depressive symptoms at baseline were also less likely to develop depression during the follow-up period. Statins are commonly prescribed to individuals who have had a cardiac event or intervention. A prospective study in this population showed that prescription of statins reduced the likelihood of developing depression by up to 79% (Stafford and Berk, 2011). A large community study also documented reduced exposure to statins and aspirin (another non-steroidal anti-inflammatory agent) in women with major depressive disorder (Pasco et al., 2010). Likewise, women who were exposed to these agents were also less likely to develop depression over the course of the study. Similar results were also observed in a large population-based cohort of elderly patients, with statins exerting a protective effect against the development of depressive symptoms (Feng et al., 2008). Notably, this study also documented a positive correlation between the use of systemic corticosteroids and depression.

The cyclooxygenase-2 (COX-2) inhibitor celecoxib is a non-steroidal anti-inflammatory drug used widely in the treatment of pain, particularly related to arthritic conditions. This drug has been found to improve depressive symptoms when administered in conjunction with the antidepressants sertraline (Abbasi et al., 2012), reboxetine (Muller et al., 2006), and fluoxetine (Akhondzadeh et al., 2009). However, it should be noted that other trials have resulted in conflicting findings, with several showing no beneficial effect of celecoxib in depression (Musil et al., 2011; Fields et al., 2012). The discrepancies in these study results are potentially reflective of the complexity of the inflammatory pathways, in which COX-2 and many other key molecules may play multiple roles. In the brain, COX-2 has anti-inflammatory and neuroprotective effects (Minghetti, 2004), and COX-2 deficient mice show increased neuronal damage, microglial reactivity and oxidative stress markers (Aid et al., 2008). Hence targeting of inflammatory pathways in depression requires careful investigation of both peripheral and central responsivity. COX-2, in particular, may not be the most appropriate target for adjunct therapies in depression [reviewed in Maes (2012)]. In addition, modulation of immune and inflammatory signaling necessitates caution with regard to the potential of lowering defenses to opportunistic infection and malignancy. Long term use of immune-modifying drugs has been associated with increased incidence of serious infections and cancer (Bongartz et al., 2006; Atzeni et al., 2012; van Dartel et al., 2013). This raises the possibility that agents which directly regulate the CNS rather than peripheral inflammatory response, or have more mild anti-inflammatory effects, may be more appropriate targets for the pharmacotherapy of depression.

Still peripherally-active, but arguably milder in effect, are the non-steroidal anti inflammatory medications, including aspirin. Animal studies using aspirin have shown moderate but discernible effects on depressive behavior (Brunello et al., 2006; Wang et al., 2011). Preliminary clinical trials have correlated this, showing a synergistic effect of co-therapy with antidepressants and aspirin (Mendlewicz et al., 2006). However, perhaps more compelling is the result from a large-scale longitudinal cohort study, which documented an association between aspirin use and lowered risk of depression (Pasco et al., 2010). Echoing this is a cross-sectional study which demonstrated that men with elevated plasma homocysteine, a marker of cardiovascular risk, had a reduced risk of depression if they had been taking aspirin (Almeida et al., 2012).

Minocycline, a second-generation tetracycline derivative, has recently attracted significant attention for its potential efficacy as an antidepressant. This well characterized drug has potent anti-inflammatory and neuroprotective effects which are independent of its antibiotic efficacy (Pae et al., 2008; Dean et al., 2012). Most importantly, minocycline readily crosses the blood brain barrier and is known to inhibit microglial activation (Pae et al., 2008; Dean et al., 2012). Studies in mice have demonstrated that minocycline attenuated the elevations in CNS IL-1β, IL-6, and IDO induced by bacterial endotoxins (Henry et al., 2008). This study also showed that pre-treatment with minocycline prevented the development of depressive-like behavioral endophenotypes, and normalized the kynurenine/tryptophan ratio in the plasma and brain (Henry et al., 2008). These findings clearly indicate that minocycline has effects on microglia through inhibition of the synthesis of pro-inflammatory cytokines and IDO up-regulation, and that these may flow through to ameliorate mood states. Echoing this, a small open-label study reported minocycline (150 mg/kg/day) in combination with serotonin reuptake inhibitor contributed to ameliorate depressive mood and psychotic symptoms in patients with psychotic unipolar depression (Miyaoka et al., 2012).

The developing appreciation of the role of inflammatory function in depression has highlighted the potential role of dietary sources of anti-inflammatory species. Deficiencies of the anti-oxidant and anti-inflammatory Coenzyme Q10 (CoQ10) have been associated with depressed mood (Maes et al., 2009), and a preliminary study of supplementation with CoQ10 showed an amelioration of depression scores in a cohort with bipolar disorder (Forester et al., 2012). Several studies in pre-clinical models have shown potential antidepressant effects of omega 3 fatty acids (Watanabe et al., 2004), and conversely, deficient diets during prenatal development have been associated with persistent changes in mood state (Chen and Su, 2013). Compounding this, altered lipid profiles have been described in the cortex of patients with mood disorders (Tatebayashi et al., 2012). Large-scale population assays have shown associations between dietary lipid profiles and the risk of depression (Hoffmire et al., 2012). Although the outcomes of clinical trials using omega 3 supplementation are still under some debate, recent meta-analyses have pointed to some degree of improved outcome in depressed patients (Lin and Su, 2007; Bloch and Hannestad, 2012; Martins et al., 2012). Intriguingly, omega 3 fatty acids have received particular attention for the treatment of depressive symptoms post-myocardial infarction (Gilbert et al., 2013; Siddiqui and Harvey, 2013). In such cases, the anti-inflammatory effects of this lipid may be ameliorating both the peripheral inflammatory state and the secondary central inflammation.

## Interfaces between HPA axis and immune dysfunction

Whilst it is clear that both inflammation and HPA dysfunction are associated with psychiatric pathology, these two systems interact at multiple levels and may together constitute a synergistic effect on neuronal function. Across the spectrum of systemic disorders associated with peripheral inflammation and an increased risk of depression, many are also associated with elevated susceptibility to, or worsening symptoms in response to stress. A large scale longitudinal study showed an association between inflammatory bowel disease (Crohn's disease and ulcerative colitis) and depressive symptoms (Ananthakrishnan et al., 2013b). These disorders are strongly associated with perceived life stress, with time to relapse predicted by stress levels (Triantafillidis et al., 2013). Studies of metabolic syndrome, diabetes and associated cardiovascular diseases have shown that not only is this suite of disorders associated with increased risk of depression and a low-grade inflammatory state, but that chronic stress is a strong promoting factor [reviewed in Kyrou and Tsigos (2009)]. These interactions may have developmental antecedents, with exposure to a high fat diet in early life being associated with both altered HPA axis function, inflammatory regulation and disordered behavioral profiles in later life (Sasaki et al., 2013). Nonetheless, the question remains as to how these complex systems interact in both the periphery and CNS, and by what mechanisms these systems modulate neuronal function and mood.

Synthetic glucocorticoids are used therapeutically at supraphysiological levels for their anti-inflammatory effects. However, when examining the relationship between the HPA axis and the immune system in physiological or pathophysiological states, the situation appears more complex. Glucocorticoids modulate the immune system through binding to receptors expressed by immune cells, which down-regulates transcription of pro-inflammatory genes and up-regulates production of anti-inflammatory cytokines (Barnes, 2006; Leonard, 2006). Glucocorticoids also regulate the circulating numbers, tissue distribution and activity profile of lymphocytes in a time-dependent manner [comprehensively reviewed in Dhabhar (2009)]. Compared to acute stress, chronic stress appears to suppress some of the protective aspects of immune regulation, whilst enhancing the drive to a pro-inflammatory state. The complexity of these interactions is reflective of the fact that chronic stimulation of the HPA axis may not in fact result in a hypercortisolaemic state; given the capacity of the HPA axis for negative feedback regulation, the baseline cortisol levels in chronic stress may actually be lower than normal. Glucocorticoids can be used therapeutically as immuno-suppressants but in some experimental models appear to have pro-inflammatory effects. Part of this discrepancy may come from differences between *in vivo* and *in vitro* models, however in addition the complexities of chronic stress in an animal model should not be overlooked. Chronic stress may appear to increase or decrease circulating glucocorticoids depending on the method of stress and the method of glucocorticoid measurement employed. An animal with chronic down-regulation of HPA axis responsivity, for example, may respond to the acute stress of blood collection or some forms of euthanasia with an overshoot of normal glucocorticoid response, giving the impression of elevated circulating hormone levels in response to the chronic stress.

Immune activation may also feed back to modulate glucocorticoid sensitivity. Production of cytokines also up-regulates expression of the GR and modulates the sensitivity of the HPA axis to negative feedback (Arzt et al., 2000). Elevation of pro-inflammatory cytokines, including IL-2, appears to inhibit nuclear translocation of the GR and suppress glucocorticoid signaling (Goleva et al., 2009; Schewitz et al., 2009). Likewise, administration of IL-1 up-regulates HPA axis activity (Dunn, 2000). Systemic exposure to pro-inflammatory stimuli such as bacterial LPS induces secretion of CRH, therefore activating the HPA axis (Sternberg, 2006). These studies illustrate the complex bidirectional interactions between HPA axis function and regulation of inflammation. Potential sex-differences have been suggested when assessing the effects of LPS on stress response. Indeed, female rats showed a higher LPS-induced corticosterone release compared to male animals (Tonelli et al., 2008). The relationship between HPA axis activity and inflammation may also be regionally specific. The peripheral response to stress and HPA activation is likely to be qualitatively, quantitatively and temporally distinct from that observed in the CNS. In a mouse model of chronic stress, increases in basal inflammatory markers were observed in multiple brain regions (Barnum et al., 2012). Chronic unpredictable stress can also up-regulate the response to peripheral inflammatory stimuli, mediated by glucocorticoid signaling (Munhoz et al., 2006). This differs somewhat to the concept of glucocorticoid signaling as immunosuppressive, and highlights the need for further investigation of the nexus between HPA and immune function in the brain. Microglia represents the critical interface point between the activity of the HPA axis, circulating inflammatory signals and the brain's inflammatory response. Microglial number and morphological changes associated with activation can be increased by chronic stress in animal models (Nair and Bonneau, 2006; Tynan et al., 2010). Blockade of glucocorticoid signaling can block stress-induced sensitization of microglial inflammatory responses (Frank et al., 2011), and microglial activation can be primed by *in vivo* exposure to glucocorticoids (Nair and Bonneau, 2006) or chronic stress (Farooq et al., 2012). Within the CNS, the balance between pro- and anti-inflammatory responses to peripheral immune stimuli is modulated by the density of microglial cells (Pintado et al., 2011).

The relationship between microglial activation and the stress response has been most comprehensively investigated in animal models. Repeated exposure to restraint stress induced microglial activation in male C57BL/6 mice, as measured by the degree of proliferation of microglia (Nair and Bonneau, 2006). The increase in microglial number was positively correlated with elevation of serum corticosterone levels induced by stress exposure. Similarly, chronic restraint stress caused a significant increase in activated microglia and number of microglia in multiple brain regions (Tynan et al., 2010; Hinwood et al., 2012), and inescapable stress potentiates the microglial response to immune stimuli (Frank et al., 2012). However, high doses of glucocorticoid agonists suppress the microglial production of inflammatory cytokines (Chantong et al., 2012). These differential responses may be reflective of central vs. peripheral differences, in addition to switching from a pro- to anti-inflammatory response to physiological vs. pharmacological levels of glucocorticoids. Nonetheless, the consensus from these studies is that microglia are acutely sensitive to both HPA axis function and inflammatory signals, and act as an inflection point between peripheral and central responses to these stimuli. As discussed above, the activation state of the microglial population has direct effects on neuronal function, via secondary cytokine production, reactive oxygen species production, neurotoxic effects and modulation of neurotransmitter production.

## Conclusions

**Figure 1:**
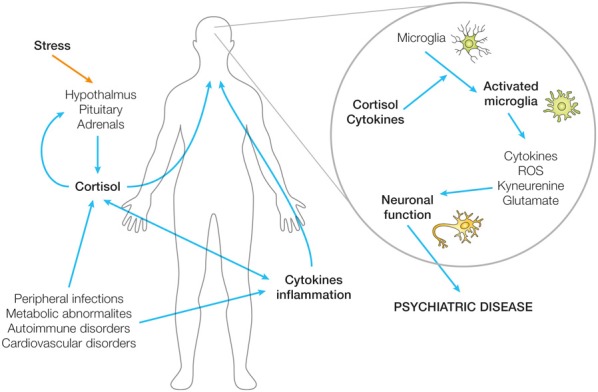
**Biological mechanisms by which peripheral dysfunction may impact on neuronal function and therefore psychiatric state**. Schematic illustration of the potential role of the CNS inflammatory cells, microglia, as a critical nexus between HPA axis activity, inflammation, and neuronal dysfunction.

It has long been established in traditional forms of medicine and in anecdotal knowledge that the health of the body and the mind are inextricably linked. Although strong associations between somatic illnesses and psychiatric disturbances have routinely been described in the literature, it is only recently that western medicine has sought to, or indeed had the means to, investigate the mechanisms underlying these associations. Strong and continually developing evidence now suggests that converging disruptions to inflammatory and endocrine pathways may interact in both the periphery and the CNS to potentiate states of psychiatric dysfunction, in particular depressed mood. Further evidence highlights the potential role of the CNS inflammatory cells, microglia, as a critical nexus between HPA axis activity, inflammation and neuronal dysfunction (Figure 1). Aspects of these pathways may therefore present as possible targets for therapeutic interventions for psychiatric disease or psychiatric complications of somatic disease. Even more efficacious may be targeting multiple aspects of these pathways or convergence points such as central microglial cells.

In this review we have focused on the biological mechanisms by which peripheral dysfunction may impact on neuronal function and therefore psychiatric state. However, we do not wish to discount the psychological influence of ill health on mental function. Clearly the psychological stresses associated with chronic illness or suboptimal health may themselves potentiate, perpetuate and exacerbate psychiatric disease. An effective clinical approach to integrated patient management therefore may need to target the HPA axis dysfunction, inflammatory changes or other pathological processes associated with peripheral disorders, but also approach the psychological health of the patient.

### Conflict of interest statement

The authors declare that the research was conducted in the absence of any commercial or financial relationships that could be construed as a potential conflict of interest.
